# Is a Wearable Sensor-Based Characterisation of Gait Robust Enough to Overcome Differences Between Measurement Protocols? A Multi-Centric Pragmatic Study in Patients with Multiple Sclerosis

**DOI:** 10.3390/s20010079

**Published:** 2019-12-21

**Authors:** Lorenza Angelini, Ilaria Carpinella, Davide Cattaneo, Maurizio Ferrarin, Elisa Gervasoni, Basil Sharrack, David Paling, Krishnan Padmakumari Sivaraman Nair, Claudia Mazzà

**Affiliations:** 1 Department of Mechanical Engineering, University of Sheffield, Sheffield S1 3JD, UK; c.mazza@sheffield.ac.uk; 2 Insigneo Institute for in silico Medicine, University of Sheffield, Sheffield S1 3JD, UK; siva.nair@nhs.net; 3 IRCCS Fondazione Don Carlo Gnocchi, 20121 Milan, Italy; icarpinella@dongnocchi.it (I.C.); dcattaneo@dongnocchi.it (D.C.); mferrarin@dongnocchi.it (M.F.); egervasoni@dongnocchi.it (E.G.); 4 Academic Department of Neuroscience, Sheffield NIHR Neuroscience BRC, Sheffield Teaching Hospital NHS Foundation Trust, Sheffield S10 2JF, UK; 5 Sheffield Institute of Translational Neuroscience, Sheffield Teaching Hospitals NHS Foundation Trust, Sheffield S10 2JF, UK

**Keywords:** multiple sclerosis, gait metrics, wearable sensors, test-retest reliability, sampling frequency, accelerometry, autocorrelation, harmonic ratio, six-minute walk

## Abstract

Inertial measurement units (IMUs) allow accurate quantification of gait impairment of people with multiple sclerosis (pwMS). Nonetheless, it is not clear how IMU-based metrics might be influenced by pragmatic aspects associated with clinical translation of this approach, such as data collection settings and gait protocols. In this study, we hypothesised that these aspects do not significantly alter those characteristics of gait that are more related to quality and energetic efficiency and are quantifiable via acceleration related metrics, such as intensity, smoothness, stability, symmetry, and regularity. To test this hypothesis, we compared 33 IMU-based metrics extracted from data, retrospectively collected by two independent centres on two matched cohorts of pwMS. As a worst-case scenario, a walking test was performed in the two centres at a different speed along corridors of different lengths, using different IMU systems, which were also positioned differently. The results showed that the majority of the temporal metrics (9 out of 12) exhibited significant between-centre differences. Conversely, the between-centre differences in the gait quality metrics were small and comparable to those associated with a test-retest analysis under equivalent conditions. Therefore, the gait quality metrics are promising candidates for reliable multi-centric studies aiming at assessing rehabilitation interventions within a routine clinical context.

## 1. Introduction

Multiple sclerosis (MS) is a chronic demyelinating disease of the central nervous system affecting 2.3 million people worldwide [1]. MS is the major non-traumatic cause of disability in young and middle-aged adults [2], with a significant negative impact on independence and social participation [3]. Walking impairment is one of the most common functional deficits due to MS, even in the early stages of the disease [4]. Importantly, nearly 70% of people with MS (pwMS) reported that walking difficulty is the most challenging aspect of their condition [5].

Given the high impact of gait impairment on pwMS, different rehabilitation interventions focused on improving locomotion are currently applied to improve the quality of life in this population [6]. The effects of these interventions, together with the progression of the disease, are usually assessed in clinical practice using clinical scales, such as the expanded disability status scale (EDSS) [7] or timed tests, such as the timed up and go test (TUG) [8], the timed 25-foot walk test (T25FW) [9], and the 6-minute walk test (6MWT) [10]. Although widely used, these tests suffer from some limitations. Firstly, they assess only the time taken to execute the test (e.g., TUG and T25FW) or the distance travelled in a given time (6 min for the 6MWT), without providing objective measures of the different components and characteristics of the task that could be useful to describe *how* the performance is possibly impaired [11]. Secondly, these clinical tests have a relatively limited sensitivity to change [9,12,13] and a flooring effect [9,14] that makes it difficult to detect possible alterations in minimally impaired pwMS [15,16,17].

Instrumental methods may partly overcome these limitations by providing additional quantitative information for a more complete characterisation of walking, which can be useful to tailor the rehabilitative intervention and objectively assess its effects [11,18]. In particular, wearable inertial measurement units (IMUs), including accelerometers, gyroscopes, and magnetometers, represent cost-effective tools to perform objective assessments of walking in pwMS outside movement analysis labs [19,20], and even during free-living and community contexts [21,22]. IMUs have been widely used to analyse different locomotor tasks in pwMS, such as straight-line over ground [17,23,24,25,26,27] and treadmill walking [28], standing up, walking, turning, and sitting down (e.g., the TUG) [15,29], walking with head turns and over/around obstacles [30,31], walking while texting [32], and stairway walking [33]. During these tests, several parameters have been extracted from IMUs, including spatio-temporal parameters [15,24,27,28,31,32,34], indexes of gait variability and stability [17,23,24,26,31,33], trunk sway metrics [15,23,30,34], and angular variables [15,25,27,34]. Nonetheless, what does not yet clearly emerge from current literature on pwMS is which of these could be more reliably adopted within the clinical context.

Besides the issue of identifying among the above metrics those that are more capable of characterising the disease progression, hence providing similar results for patients with similar clinical conditions, and that have the sensitivity to detect changes associated with clinical interventions, the clinical adoption of specific gait metrics also requires accounting for a number of pragmatic limitations associated with testing conditions. These include an understanding of which output is more robust to testing site characteristics (e.g., corridor lengths, lightening, noise, etc.), adopted measuring instruments and their configuration (e.g., brand, location on the body, sampling frequency) [35,36,37], type of gait test (e.g., a single pass, a 1-minute or a 6MWT), or instructions given to patients (e.g., self-selected or fast walking speed, use or not use of an assistive device) [28,38,39,40,41,42,43,44,45]. All these aspects are particularly difficult to standardise in a busy clinical environment and most likely occur in combination with each other.

The aim of this study was to identify those gait metrics that provide equivalent assessment of pwMS with similar characteristics in terms of age, gender, and gait disability, despite these being tested in different centres and in non-standardised conditions. Our hypothesis was that while pwMS might be able to adjust their gait in terms of spatio-temporal parameters in response to different testing conditions (e.g., if asked to increase their speed), they would not be able to control those aspects of gait more related to its overall quality and energetic efficiency [46,47]. As a result, metrics extracted directly from the acceleration signals and representative of intensity, smoothness, stability, symmetry, and regularity were expected to be more robust to differences in the test settings. To verify this hypothesis, we compared retrospective data from two matched cohorts of pwMS, which were collected by two independent hospitals using protocols that differed for: (i) brand, size, and sampling frequency of the IMUs; (ii) IMU positioning; (iii) subject instructing; (iv) length of the path. As a term of reference, we also compared differences in IMU-based metrics between the two centres (between-centre differences) to those observable between two sessions performed by the same centre (between-day test-retest reliability).

## 2. Materials and Methods

### 2.1. Participants

Two research centres, one located in Italy (centre A) and one in the United Kingdom (centre B), provided retrospective IMU data collected while pwMS walked back and forth for 6 min along a hospital corridor. The patients’ level of disability was assessed with the EDSS scale, scored by an experienced neurologist. Patients were excluded if not free from any orthopaedic and/or musculoskeletal and neurological disorders other than MS that may have affected their gait and balance. Since there were no restrictions for MS subtypes, both patients with relapsing remitting MS who were relapse-free for 30 days prior to assessment (centre A) and patients with secondary progressive MS (centre B) were included in the study. Thirteen pwMS were selected from each data set to form two cohorts, with individual patients matched if having the same age, gender, EDSS score, and type of assistive device (Table 1). As a result of this matching, the sample size, percentage of females, EDSS score distribution, number of pwMS who required an assistive device, and type of assistive device used during the walking test were the same in the two centres. The average walking speed was calculated as the total distance walked during the test divided by the duration of the walking trial.

pwMS from centre B repeated the instrumented walking test on a second visit, which was held 7–14 days after the first test at the same time of the day. The testing procedures were also kept constant between the two sessions. These data were used to assess between-day test-retest reliability.

Institutional review boards or ethics committees at the institutions in each country approved the separate protocols (NRES Committee Yorkshire & The Humber-Bradford Leeds (reference 15/YH/0300) and Ethical Committee of Don Carlo Gnocchi Foundation, Milan, Italy, references 29-03-2017 and 13-02-2019). Written informed consent was provided by all subjects. Data were collected in accordance with the International Declaration of Helsinki.

### 2.2. Experimental Protocol

Acceleration and angular velocity data from three IMUs, located at the fifth lumbar vertebra and around the right and left ankles, were recorded in both centres while pwMS walked back and forth for 6 min along a straight corridor free of obstacles and other people. If needed, they could use an assistive device and take short resting breaks while standing. Each IMU was manually aligned along the anatomical antero-posterior (AP), medio-lateral (ML), and vertical (V) axes.

**Figure 1:**
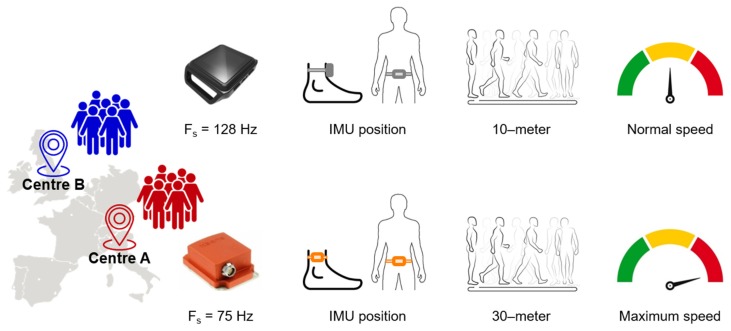
Experimental protocols followed by centre A (red) and centre B (blue).

The differences between the experimental protocols followed by centre A and centre B were: (i) device manufacturers and sampling frequency used to record acceleration and angular velocity signals; (ii) ankle IMU position; (iii) length of the walkway; (iv) instructions given to participants (Figure 1). Specifically, Xsens IMUs (unit weight 16 g, unit size 47 mm  ×  30 mm  ×  13 mm; MTw, Xsens, NL) with a sampling frequency of 75 Hz were used in centre A and OPAL IMUs (unit weight 22 g, unit size 48.5 mm  ×  36.5 mm  ×  13.5 mm; OPAL, APDM Inc., Portland, OR, USA) with a sampling frequency of 128 Hz were used in centre B. The IMUs around both ankles were placed laterally in centre A and frontally in centre B. PwMS were requested to walk at their maximum speed along a 30-meter straight corridor in centre A and at preferred comfortable speed along a 10-meter straight corridor in centre B.

### 2.3. Data Processing

Data processing routines were developed in Matlab^®^ (MATLAB R2019b, MathWorks, Inc., Natick, MA, USA). A total of 33 IMU-based metrics were included in this analysis. IMU signals collected in centre B were down sampled from 128 Hz to 75 Hz to match data from centre A, and the influence of down sampling was investigated by comparing the outcome metrics from centre B as obtained before and after the down sampling. Data from the lumbar IMU were reoriented to a horizontal-vertical coordinate system [48] and filtered with a 10 Hz cut-off, zero phase, low-pass Butterworth filter.

The turning motion and resting breaks were detected and removed from IMU signals to isolate steady-state walking bouts, which were used to compute the metrics of interest. The approach proposed by Salarian, et al. [49] was adapted to determine 180° turns, which appear in the V component of the lumbar angular velocity, *ω_z_(t)*, as peaks of a given duration. The turning onset and offset were identified from the trunk rotation angle around the V axis, *θ_z_(t)*, obtained after integrating the *ω_z_(t)* signal. The turning components were evidenced in *θ_z_(t)* as steep positive or negative gradients, whereas walking components were evidenced as small oscillations round a flat line. Specifically, *θ_z_(t)* was first smoothed using a weighted least-squares linear regression. Abrupt change points and their locations were then searched in *θ_z_(t)* using a predefined Matlab^®^ function based on the minimisation of a linear computational cost function [50]. Resting breaks were automatically detected by checking in 2-s window increments if: (i) the norm of the lumbar IMU angular velocity was less than 0.5 rad/s; (ii) the norm of the lumbar IMU acceleration was within ±10% of 9.81 m/s² [51]. A 2-s window was considered motionless if more than 50% of its samples fulfilled both criteria mentioned above.

Twelve gait metrics were extracted from the angular velocities recorded from the ankle IMUs and 21 were extracted from the lumbar IMU accelerations. Following the suggestions of Lord, et al. [52] and Buckley, et al. [53], these metrics were organised in independent gait domains (e.g., rhythm, variability, asymmetry, intensity, stability, smoothness, symmetry, and regularity).

Initial and final foot contact instances, referred to as gait events (GE), were identified for each steady-state walking bout as local minimum values of the ML angular velocity recorded from ankle IMUs of both legs [54]. These minima occur just before and after the instant of maximum ML angular velocity. Once the GE were determined, stride, step, swing and stance durations (representing rhythm domain) were separately estimated for left and right sides. Variability (i.e., within-subject combined standard deviation of left and right; variability domain) and asymmetry (i.e., absolute difference between the mean of left and right time series; asymmetry domain) of these metrics were also computed, applying the established formula in Galna, et al. [55] and Godfrey, et al. [56]. 

From processing the filtered acceleration signals in time and frequency domain, 21 additional metrics, referred to as gait quality metrics [57], were separately extracted for each acceleration component (AP, ML, and V): (i) intensity as the root mean square (RMS) of each acceleration component around its mean value [44]; (ii) stability as the ratio of the RMS in a given direction to the RMS vector magnitude [58]; (iii) smoothness as the RMS of the jerk [59]; (iv) symmetry represented by the harmonic ratio (HR), defined as the ratio of the sum of the amplitudes of the in-phase harmonics to the sum of the amplitudes of the out-of-phase harmonics [60,61]; (v) regularity as the ensemble of the following three metrics obtained from the unbiased normalised autocorrelation [62]:

(1) $$Step\;regularity=1st\;peak\;of\;\left(\frac{1}{N-\left|m\right|}\overset{N-\left|m\right|}{\underset{i=1}{\sum }}x\left(i\right)\cdot x\left(i+m\right)\right)$$

(2) $$Stride\;regularity=2nd\;peak\;of\;\left(\frac{1}{N-\left|m\right|}\overset{N-\left|m\right|}{\underset{i=1}{\sum }}x\left(i\right)\cdot x\left(i+m\right)\right)$$

(3) $$Regularity\;index=\frac{\left|Stride\;regularity-Step\;regularity\right|}{mean\left(Stride\;regularity,Step\;regularity\right)}$$

All metrics were calculated for the part of signals corresponding to the middle eight steps of each pass along the corridor and then averaged over the whole trial. The choice of eight steps was due to the maximum number of steps which subjects in centre B could walk in completely straight condition. Since centre A adopted a three-times longer path, in order to process the same number of steps, only one walking bout in every three was included for centre B.

### 2.4. Statistical Analysis

Statistical analyses were performed in R version 3.4.3 [63]. Participant characteristics from centre A and centre B were compared using the independent Mann-Whitney U for age and EDSS scores and Pearson’s chi-square for gender. Given the limited sample size and the non-normal distribution of most of the investigated metrics (as a result of the Shapiro-Wilk test), non-parametric tests were performed. The level of significance was taken at 5%. A Wilcoxon signed-rank test was performed to compare the centre B metrics obtained from IMU data sampled at 128 Hz and those down-sampled at 75 Hz.

Between-day test-retest reliability of the metrics was evaluated for centre B through the intra-class correlation coefficients (ICCs) with a 95% confidence interval (CI). ICCs were calculated using a two-way random-effect model and absolute agreement (ICC2,k) [64]. An ICC lower than 0.39 was classified as poor, an ICC between 0.40 and 0.59 as fair, an ICC between 0.60 and 0.74 as moderate, and an ICC greater than 0.75 as excellent [65]. The minimum detectable changes (MDCs), representing the smallest amount of change that can be considered above the bounds of the measurement error and/or within-subject variability, was also computed for each metric at the CI of 95%, according to Equation (4):

(4) $$MDC=1.96\cdot \sqrt{2}\cdot SEM=1.96\cdot \sqrt{2}\cdot SD\cdot \sqrt{1-ICC},$$

where SEM is the standard error of the measurement and SD corresponds to the average of the standard deviations from test and re-test sessions [66].

A Wilcoxon signed-rank test was used to determine if there was a median difference in centre B metrics between the two sessions, whereas an independent Mann-Whitney U test was carried out to compare IMU-based metrics from centre A and centre B. 

In all the above tests, if the *p*-value was lower than 0.05, the null hypothesis (e.g., the two population medians were identical) was rejected and the alternative hypothesis accepted. To avoid misinterpretation of the *p*-values and to account for a type II error, the effect size (*r*) for non-parametric tests was also calculated as follows:

(5) r=zN

where z is the z-score and N is the size of the study (i.e., the number of total observations) on which z is based. Cohen [67] suggested thresholds of 0.1, 0.3, and 0.5 for small, medium, and large effect sizes, respectively.

Median, inter-quartile range, minimum, and maximum values were finally calculated for IMU-based metrics from centre A and centre B (both sessions).

## 3. Results

### 3.1. Effect of Sampling Frequency

The results of the comparison between the metrics calculated using the 128 Hz and 75 Hz sampling frequencies are reported in Table 2. The HR, representative of the symmetry domain, was the only metric that significantly differed between the two analyses.

### 3.2. Between-Day Test-Retest Reliability

**Figure 2:**
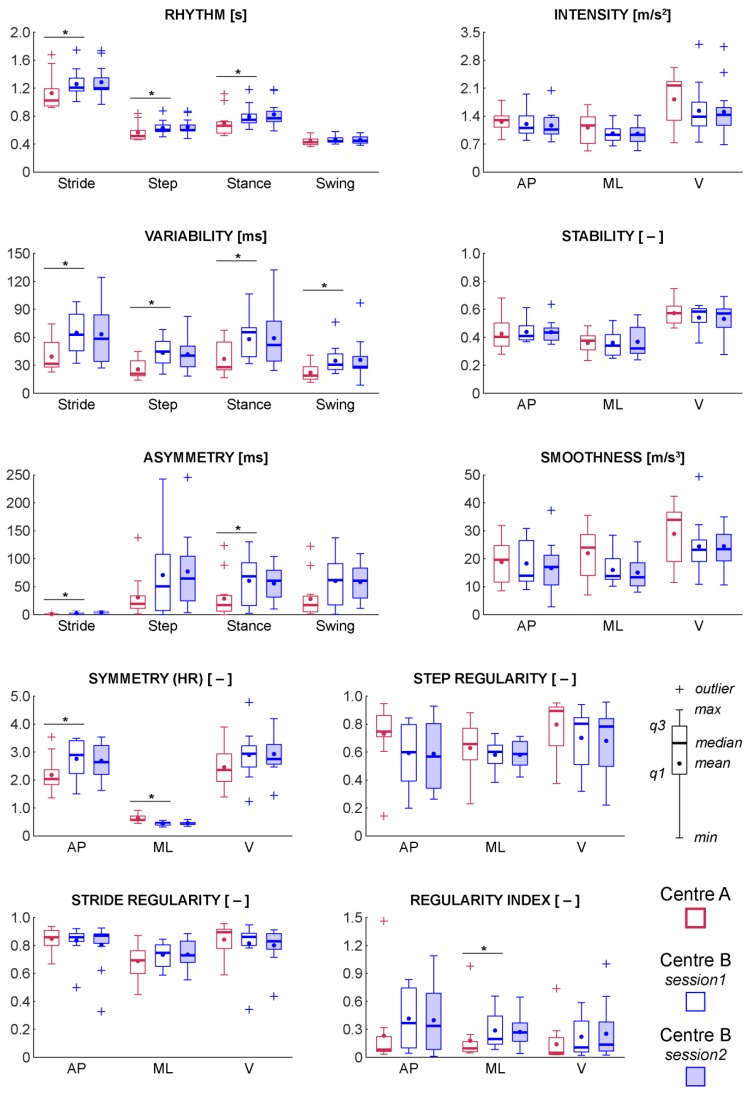
Minimum, first quartile (q1), median, mean, third quartile (q3), and maximum values of each IMU-based metrics relative to centre A (red) and centre B for between-day test-retest assessment (blue empty boxplots and blue filled boxplots). Values larger than q1 + 1.5(q3 + q1) or smaller than q1 − 1.5(q3 − q1) are considered outliers and are represented with crosses (+). * *p* < 0.05. Note that, for graphical convenience, the absolute values have been depicted for the step regularity and regularity index in the ML direction.

ICC, SEM, and MDC values for between-day assessment are shown in Table 3 for each metric estimated for pwMS from centre B who completed two testing visits. Overall, 17 out of 33 metrics revealed excellent test-retest reliability (ICC: 0.93–0.98; 95% CI: 0.76–0.93), 11 metrics showed moderate test-retest reliability (ICC: 0.88–0.92; 95% CI: 0.62–0.74), and only 5 metrics exhibited poor to fair test-retest reliability with ICC values between 0.72 and 0.86 and 95% CI between 0.13 and 0.52. The Wilcoxon signed-rank test showed no significant differences in any of the metrics between the two sessions (Figure 2 and Table 4).

### 3.3. Between-Centre Differences

As expected, the comparison between centre A and centre B via the independent Mann-Whitney U test highlighted significant differences for all the temporal metrics (Figure 2 and Table 5; rhythm domain), except for swing duration. Apart from asymmetry of step duration and asymmetry of swing duration, variability and asymmetry of the temporal metrics were significantly lower in centre A compared to centre B (Figure 2 and Table 5; variability and asymmetry domain). However, even though the difference in asymmetry of swing duration between the two centres was non-significant (U = 48.0; *p* = 0.06), a fairly moderate effect size was found for this specific metric (*r* = 0.37). Conversely, a consistency between the two centres was found for 18 out of 21 metrics extracted from acceleration signals (Figure 2 and Table 5; intensity, stability, smoothness, symmetry, and regularity domains). Only the differences in the regularity index in the ML direction and in the HR in the AP and ML directions were proved statistically significant between centre A and centre B (Figure 2 and Table 5).

## 4. Discussion

This study aimed to identify comparable gait metrics as quantified from IMU data measured from two different hospital settings on two matched cohorts of pwMS (13 pwMS for each centre, Table 1), under the hypothesis that those metrics associated with the overall balance control and coordination of gait (i.e., gait quality metrics) would be robust, even when obtained from different experimental protocols. Reported results overall corroborated this assumption and showed that between-centre differences for most of these metrics were comparable to those obtained by the same centre in two different sessions.

The small sample size, resulting from the attempt of maximising the cohort match, is certainly a limitation of this study. It is worth noting, in fact, that while some of the investigated gait metrics in centre A (e.g., asymmetry of swing duration from asymmetry domain and regularity index from regularity domain) did not differ significantly from those in centre B, an observed medium effect size suggested the opposite might hold true (Table 5). This is indeed likely to be due to the small sample size and possibly due to the higher inter-subject variability observed in centre B.

Since MS is well known for heterogeneity of symptoms, high day-to-day fluctuations, and a large variability in its course [68], care must be taken before generalising our findings to all pwMS with different levels of gait impairment. Another limitation of this study might lie in the fact that patients recruited by the two centres differed in the subtypes of MS. Nonetheless, Dujmovic, et al. [69] showed that the altered gait pattern in pwMS did not depend on the disease phenotype. Additional studies are of course needed to further investigate this aspect.

The comparison between centre A and centre B implied down-sampling the data from the latter. As expected, this affected only the calculation of HR, which is the only metric based on frequency analysis. In particular, changing sampling frequency from 128 Hz to 75 Hz led to decreased values in the AP and V directions and increased values in the ML direction (Table 2). This is in line with what was previously reported by Riva, et al. [35].

Moderate to excellent between-day test-retest reliability was observed for 28 out of 33 IMU-based metrics with few exceptions, which exhibited poor to fair reliability (Table 3). Additionally, all the investigated metrics were not significantly different between the two sessions (Figure 2 and Table 4), even if some of these results (swing duration in particular) should be interpreted with care, due to the medium effect size. These findings confirmed that sensor-based gait analysis is a reliable tool in pwMS, as also reported in previous test-retest studies on pwMS [34].

Walking speed clearly affected the gait outcomes. In particular, the gait metrics representative of rhythm, variability, and asymmetry domains were evidently lower in centre A compared to centre B (Figure 2 and Table 5) due to different instructions given to the participants in terms of walking speed (i.e., walk at maximum speed versus walk at self-selected speed). This finding is in agreement with previous studies on pwMS [28] and on people with other neurological conditions, such as Parkinson’s disease [70], which observed a reduction of the above metrics with increasing walking speed. The shorter length of the walkway used in centre B could also have contributed to these differences. In fact, Storm, et al. [22] demonstrated that rhythm and variability metrics decreased when walking longer distances (e.g., lower stride duration and lower variability of stride duration). However, the data available for our study did not allow us to separate walking speed and path effects, and further studies should hence be performed to this purpose.

Unlike the temporal metrics, the gait quality metrics appeared to be robust with respect to the notable differences in the experimental gait protocols adopted by the two centres. Among these metrics, in fact, only differences in the regularity index in the ML direction and the HR (representative of symmetry domain) in the AP and ML directions were found to be statistically significant between centre A and centre B (Figure 2 and Table 5). Again, this specific result could be explained both by the different walking speed and by the different lengths of the walkway in the two centres. Indeed, an association between walking speed and HR has been previously showed, both in healthy young [43,44] and older subjects [39]. These authors observed that the HR increased at the self-selected comfortable walking speed and decreased at slower and faster speeds. A similar trend emerged from our analysis, except for the HR in the ML direction, but this specific metric should be handled with care due to its observed low test-retest reliability (Table 3). The low number of steps (i.e., eight steps) used for calculating the HR for each walking bout might also have contributed to reduce robustness and reliability of this metric [57,71]. However, this choice was imposed by the reduced length of the corridor in centre B. Testing the participants along a shorter path also implied a higher number of turns over the 6 min, resulting in a minor validity of the HR as showed in the research by Riva, et al. [35] and by Brach, et al. [40].

While further studies are of course needed to fully validate this hypothesis, our results suggest that, in agreement with what is already reported for other neurological diseases, such as Parkinson’s disease [53], the gait quality metrics extracted from the upper body accelerations should not be considered as a simple reflection of gait spatio-temporal features and might bring complementary informative content in quantifying patients’ gait ability. Additionally, these metrics have been recently shown to be sensitive to fatigue and pathology progression in pwMS [72] and, as such, they are promising candidates for quantification of disease progression and rehabilitation interventions in these patients.

## 5. Conclusions

In conclusion, this pragmatic study showed consistency in the gait metrics from two matched groups of pwMS, even when they were assessed in two different hospitals and under notably different gait testing conditions. The identification of such robust gait metrics opens the possibility of comparing retrospective data and paves the way for reliable multi-centre studies to be conducted in routine hospital settings rather than in specialised gait research laboratories. This is essential to allow an increase of sample size and statistical power of clinical trials in which rehabilitation interventions need to be quantitatively assessed.

## Figures and Tables

**Table 1:** Clinical characteristics of people with multiple sclerosis for centre A and centre B. Abbreviations: expanded disability status scale (EDSS); people with multiple sclerosis (pwMS); Mann-Whitney U (MWU) statistic; *p*-value (*p*); chi-square (X^2^).

	Centre A (n = 13)	Centre B (n = 13)	Statistics
Age [years]	51 (35–63)	57 (34–64)	U = 58, *p* = 0.18
Gender [men/women]	3/10	3/10	X^2^(1) = 0.00, *p* = 1.00
EDSS score (0–10)	4.5 (2.0–6.5)	4.5 (2.5–6.5)	U = 83, *p* = 0.93
*Mild (2.0–2.5)*	1	1	
*Moderate (3.0–4.5)*	6	6	
*Severe (5.0–6.5)*	6	6	
Assistive devices			
*Walker*	1 pwMS	1 pwMS	–
*Cane*	2 pwMS	2 pwMS	–
Walking speed [m/s]	1.1 (0.5–1.4)	0.7 (0.4–1.0)	U = 31, *p* < 0.01 *

Values are median (range) or numbers. * *p* < 0.05.

**Table 2:** Effect of down-sampling of the acceleration and angular velocity signals on the investigated gait metrics. Abbreviations: sampling frequency (F_S_), z-score (z), *p*-value (*p*), and effect size (*r*).

Domain	F_s_ of 128 Hz	F_s_ of 75 Hz	z	*p*	*r*
**Rhythm [s]**					
Stride duration	1.20 (1.01–1.74)	1.21 (1.01–1.74)	−0.82	0.41	−0.16
Step duration	0.60 (0.51–0.87)	0.60 (0.50–0.87)	0.00	1.00	0.00
Stance duration	0.75 (0.61–1.18)	0.75 (0.61–1.18)	−1.83	0.07	−0.36
Swing duration	0.44 (0.40–0.58)	0.44 (0.40–0.58)	−1.85	0.06	−0.36
**Variability [ms]**					
Stride duration	61 (32–100)	63 (32–98)	−1.55	0.12	−0.30
Step duration	46 (20–69)	45 (20–68)	−1.33	0.18	−0.26
Stance duration	65 (34–105)	65 (32–106)	−0.18	0.86	−0.04
Swing duration	29 (23–74)	30 (21–76)	−0.41	0.68	−0.08
**Asymmetry [ms]**					
Stride duration	2 (0–7)	2 (1–7)	−0.09	0.93	−0.02
Step duration	56 (0–238)	51 (0–242)	−1.49	0.14	−0.29
Stance duration	61 (3–149)	69 (2–130)	−1.58	0.11	−0.31
Swing duration	54 (1–155)	62 (0–138)	−1.33	0.18	−0.26
**Intensity [m/s^2^]**					
Antero-Posterior	1.10 (0.80–1.96)	1.10 (0.80–1.95)	−0.94	0.34	−0.19
Medio-Lateral	0.93 (0.65–1.41)	0.93 (0.65–1.41)	−1.44	0.15	−0.28
Vertical	1.37 (0.76–3.16)	1.38 (0.75–3.20)	−1.28	0.20	−0.25
**Stability** [–]					
Antero-Posterior	0.41 (0.37–0.61)	0.41 (0.37–0.61)	−0.30	0.77	−0.06
Medio-Lateral	0.34 (0.25–0.52)	0.34 (0.25–0.52)	−0.89	0.37	−0.18
Vertical	0.58 (0.36–0.62)	0.58 (0.36–0.63)	−0.29	0.77	−0.06
**Smoothness [m/s^3^]**					
Antero-Posterior	13.97 (8.86–30.75)	13.95 (8.93–30.82)	0.00	1.00	0.00
Medio-Lateral	13.92 (10.13–28.61)	13.87 (10.19–28.45)	−1.06	0.29	−0.21
Vertical	23.31 (11.06–48.81)	23.21 (10.89–49.38)	−0.75	0.45	−0.15
**Symmetry (HR) [–]**					
Antero-Posterior	2.94 (1.49–3.73)	2.89 (1.50–3.49)	−2.32	0.02 *	−0.45
Medio-Lateral	0.44 (0.32–0.56)	0.45 (0.32–0.56)	−2.19	0.03 *	−0.43
Vertical	3.01 (1.21–4.84)	2.94 (1.23–4.78)	−3.01	0.00 *	−0.59
**Regularity [–]**					
*Step regularity*					
Antero-Posterior	0.60 (0.20–0.85)	0.60 (0.20–0.84)	−1.70	0.09	−0.33
Medio-Lateral	−0.62 (−0.74–−0.37)	−0.60 (−0.73–−0.38)	−1.89	0.06	−0.37
Vertical	0.81 (0.32–0.95)	0.80 (0.32–0.94)	−1.44	0.15	−0.28
*Stride regularity*					
Anterior-Posterior	0.86 (0.50–0.93)	0.86 (0.50–0.92)	0.00	1.00	0.00
Medio-Lateral	0.77 (0.58–0.85)	0.75 (0.59–0.85)	−1.67	0.09	−0.33
Vertical	0.86 (0.34–0.95)	0.86 (0.34–0.95)	−1.80	0.07	−0.35
*Regularity index*					
Antero-Posterior	0.37 (0.04–0.82)	0.37 (0.04–0.83)	−1.10	0.27	−0.22
Medio-Lateral	−0.20 (−0.70–−0.08)	−0.20 (−0.66–−0.08)	−0.41	0.68	−0.08
Vertical	0.11 (0.02–0.59)	0.11 (0.02–0.59)	0.00	1.00	0.00

Values are median (range). * *p* < 0.05.

**Table 3:** Intra-class correlation coefficients (ICC) with a 95% confidence interval (CI), standard error of the measurement (SEM), and minimum detectable change (MDC) for the investigated gait metrics.

Domains	ICC	95% CI		SEM	MDC
		Lower	Upper		
**Rhythm [s]**					
Stride duration	0.97	0.90	0.99	0.04	0.10
Step duration	0.97	0.90	0.99	0.02	0.05
Stance duration	0.96	0.86	0.99	0.03	0.09
Swing duration	0.97	0.91	0.99	0.01	0.03
**Variability [ms]**					
Stride duration	0.92	0.73	0.97	8	21
Step duration	0.92	0.74	0.98	5	13
Stance duration	0.94	0.80	0.98	6	18
Swing duration	0.95	0.85	0.99	4	11
**Asymmetry [ms]**					
**Stride duration**	**0.72**	**0.13**	**0.91**	**1**	**4**
Step duration	0.98	0.93	0.99	10	29
Stance duration	0.90	0.67	0.97	12	33
Swing duration	0.89	0.62	0.97	13	36
**Intensity [m/s^2^]**					
Antero-Posterior	0.97	0.90	0.99	0.06	0.16
Medio-Lateral	0.98	0.93	0.99	0.04	0.11
Vertical	0.97	0.92	0.99	0.10	0.29
**Stability [–]**					
Antero-Posterior	0.93	0.78	0.98	0.02	0.05
Medio-Lateral	0.93	0.76	0.98	0.03	0.08
Vertical	0.91	0.69	0.97	0.03	0.09
**Smoothness [m/s^3^]**					
Antero-Posterior	0.92	0.73	0.97	2.46	6.83
Medio-Lateral	0.93	0.79	0.98	1.55	4.29
Vertical	0.95	0.82	0.98	2.31	6.41
**Symmetry (HR) [–]**					
Antero-Posterior	0.95	0.85	0.99	0.14	0.38
**Medio-Lateral**	**0.75**	**0.15**	**0.92**	**0.04**	**0.10**
Vertical	0.92	0.74	0.98	0.21	0.59
**Regularity [–]**					
*Step regularity*					
Antero-Posterior	0.91	0.70	0.97	0.07	0.19
**Medio-Lateral**	**0.86**	**0.52**	**0.96**	**0.04**	**0.11**
Vertical	0.97	0.92	0.99	0.04	0.10
*Stride regularity*					
Antero-Posterior	0.88	0.64	0.96	0.05	0.13
**Medio-Lateral**	**0.85**	**0.50**	**0.96**	**0.04**	**0.10**
Vertical	0.93	0.77	0.98	0.04	0.10
*Regularity index*					
**Antero-Posterior**	**0.76**	**0.17**	**0.93**	**0.17**	**0.47**
Medio-Lateral	0.88	0.62	0.96	0.06	0.17
Vertical	0.89	0.63	0.97	0.09	0.24

Inertial measurement unit (IMU)-based gait metrics with poor to fair test-retest reliability are presented in bold.

**Table 4:** Descriptive statistics for the investigated gait metrics from centre B (session1 and session2), including the z-score (z), *p*-value (*p*), and effect size (*r*).

Domain	Centre B (session1)	Centre B (session2)	z	*p*	*r*
**Rhythm [s]**					
Stride duration	1.21 (1.01–1.74)	1.20 (0.97–1.74)	−0.70	0.48	−0.14
Step duration	0.60 (0.50–0.87)	0.60 (0.48–0.87)	−0.56	0.58	−0.11
Stance duration	0.75 (0.61–1.18)	0.77 (0.59–1.18)	−1.57	0.12	−0.31
Swing duration	0.44 (0.40–0.58)	0.45 (0.38–0.56)	−1.99	0.05	−0.39
**Variability [ms]**					
Stride duration	63 (32–98)	58 (27–124)	−0.35	0.72	−0.07
Step duration	45 (20–68)	40 (18–83)	−0.35	0.72	−0.07
Stance duration	65 (32–106)	52 (24–132)	−0.03	0.97	−0.01
Swing duration	30 (21–76)	28 (9–97)	−0.53	0.60	−0.10
**Asymmetry [ms]**					
Stride duration	2 (1–7)	4 (0–7)	−1.34	0.18	−0.26
Step duration	51 (0–242)	65 (4–245)	−1.30	0.20	−0.25
Stance duration	69 (2–130)	61 (10–104)	−0.52	0.60	−0.10
Swing duration	62 (0–138)	61 (12–109)	−0.38	0.70	−0.08
**Intensity [m/s^2^]**					
Antero-Posterior	1.10 (0.80–1.95)	1.07 (0.76–2.04)	−1.22	0.22	−0.24
Medio-Lateral	0.93 (0.65–1.41)	0.93 (0.53–1.42)	−0.08	0.94	−0.02
Vertical	1.38 (0.75–3.20)	1.43 (0.68–3.14)	−0.38	0.70	−0.08
**Stability [–]**					
Antero-Posterior	0.41 (0.37–0.61)	0.43 (0.35–0.64)	−0.28	0.78	−0.05
Medio-Lateral	0.34 (0.25–0.52)	0.32 (0.24–0.56)	0.00	1.00	0.00
Vertical	0.58 (0.36–0.63)	0.57 (0.28–0.69)	−0.27	0.79	−0.05
**Smoothness [m/s^3^]**					
Antero-Posterior	13.95 (8.93–30.82)	17.11 (2.76–37.32)	−1.17	0.24	−0.23
Medio-Lateral	13.87 (10.19–28.45)	13.42 (8.04–26.07)	−1.24	0.22	−0.24
Vertical	23.21 (10.89–49.38)	23.43 (10.67–50.74)	−0.41	0.68	−0.08
**Symmetry (HR) [–]**					
Antero-Posterior	2.89 (1.50–3.49)	2.64 (1.62–3.54)	−0.31	0.75	−0.06
Medio-Lateral	0.45 (0.32–0.56)	0.46 (0.34–0.59)	−0.82	0.41	−0.16
Vertical	2.94 (1.23–4.78)	2.75 (1.45–4.19)	−0.51	0.61	−0.10
**Regularity [–]**					
*Step regularity*					
Antero-Posterior	0.60 (0.20–0.84)	0.57 (0.26–0.93)	−0.12	0.91	−0.02
Medio-Lateral	−0.60 (−0.73–−0.38)	−0.58 (−0.71–−0.42)	−0.07	0.94	−0.01
Vertical	0.80 (0.32–0.94)	0.78 (0.22–0.96)	−1.26	0.21	−0.25
*Stride regularity*					
Anterior-Posterior	0.86 (0.50–0.92)	0.87 (0.33–0.92)	−0.98	0.33	−0.19
Medio-Lateral	0.75 (0.59–0.85)	0.73 (0.55–0.88)	−0.03	0.97	−0.01
Vertical	0.86 (0.34–0.95)	0.83 (0.44–0.91)	−0.52	0.60	−0.10
*Regularity index*					
Antero-Posterior	0.37 (0.04–0.83)	0.34 (0.01–1.09)	−0.04	0.97	−0.01
Medio-Lateral	−0.20 (−0.66–−0.08)	−0.27 (−0.65–−0.04)	−0.14	0.89	−0.03
Vertical	0.11 (0.02–0.59)	0.14 (0.02–1.00)	−0.43	0.67	−0.08

Values are median (range). * *p* < 0.05.

**Table 5:** Descriptive statistics for the investigated gait metrics from centre A and centre B (session1), including the Mann-Whitney U (MWU) statistic, *p*-value (*p*), and effect size (*r*).

Domain	Centre A	Centre B	U	*p*	*r*
**Rhythm [s]**					
Stride duration	1.03 (0.92–1.68)	1.21 (1.01–1.74)	43.5	0.04 *	0.41
Step duration	0.51 (0.46–0.84)	0.60 (0.50–0.87)	44.0	0.04 *	0.41
Stance duration	0.66 (0.52–1.12)	0.75 (0.61–1.18)	40.0	0.02 *	0.45
Swing duration	0.43 (0.37–0.56)	0.44 (0.40–0.58)	57.0	0.17	0.28
**Variability [ms]**					
Stride duration	32 (23–74)	63 (32–98)	26.0	0.00 *	0.59
Step duration	21 (14–45)	45 (20–68)	27.5	0.00 *	0.57
Stance duration	28 (17–68)	65 (32–106)	30.0	0.01 *	0.55
Swing duration	19 (12–41)	30 (21–76)	33.0	0.01 *	0.52
**Asymmetry [ms]**					
Stride duration	1 (0–4)	2 (1–7)	45.0	0.04 *	0.40
Step duration	19 (1–138)	51 (0–242)	68.0	0.40	0.17
Stance duration	17 (0–123)	69 (2–130)	46.0	0.04 *	0.39
Swing duration	17 (1–122)	62 (0–138)	48.0	0.06	0.37
**Intensity [m/s^2^]**					
Antero-Posterior	1.30 (0.81–1.80)	1.10 (0.80–1.95)	68.0	0.41	0.17
Medio-Lateral	1.17 (0.53–1.69)	0.93 (0.65–1.41)	62.0	0.26	0.23
Vertical	2.17 (0.73–2.62)	1.38 (0.75–3.20)	59.5	0.21	0.25
**Stability [–]**					
Antero-Posterior	0.40 (0.28–0.68)	0.41 (0.37–0.61)	69.0	0.44	0.16
Medio-Lateral	0.38 (0.23–0.48)	0.34 (0.25–0.52)	83.0	0.96	0.02
Vertical	0.57 (0.47–0.75)	0.58 (0.36–0.63)	72.5	0.55	0.12
**Smoothness [m/s^3^]**					
Antero-Posterior	19.68 (8.56–31.85)	13.95 (8.93–30.82)	82.0	0.92	0.03
Medio-Lateral	24.01 (7.04–35.54)	13.87 (10.19–28.45)	52.5	0.11	0.32
Vertical	33.90 (11.56–42.39)	23.21 (10.89–49.38)	59.0	0.20	0.26
**Symmetry (HR) [–]**					
Antero-Posterior	2.04 (1.36–3.54)	2.89 (1.50–3.49)	43.5	0.04 *	0.41
Medio-Lateral	0.57 (0.44–0.91)	0.45 (0.32–0.56)	14.0	0.00 *	0.71
Vertical	2.35 (1.39–3.89)	2.94 (1.23–4.78)	52.5	0.11	0.32
**Regularity [–]**					
*Step regularity*					
Antero-Posterior	0.75 (0.14–0.95)	0.60 (0.20–0.84)	55.5	0.14	0.29
Medio-Lateral	−0.66 (−0.88–−0.23)	−0.60 (−0.73–−0.38)	63.0	0.28	0.22
Vertical	0.89 (0.37–0.95)	0.80 (0.32–0.94)	51.5	0.10	0.33
*Stride regularity*					
Anterior-Posterior	0.86 (0.67–0.93)	0.86 (0.50–0.92)	81.5	0.90	0.03
Medio-Lateral	0.69 (0.45–0.87)	0.75 (0.59–0.85)	63.0	0.28	0.22
Vertical	0.89 (0.59–0.96)	0.86 (0.34–0.95)	72.5	0.55	0.12
*Regularity index*					
Antero-Posterior	0.08 (0.03–1.46)	0.37 (0.04–0.83)	50.0	0.08	0.35
Medio-Lateral	−0.10 (−0.98–−0.05)	−0.20 (−0.66–−0.08)	40.5	0.03 *	0.44
Vertical	0.05 (0.02–0.74)	0.11 (0.02–0.59)	51.5	0.09	0.33

Values are median (range). * *p* < 0.05.

## Data Availability

The data used in this paper will be made publicly available (DOI: 10.15131/shef.data.11395641).
